# Optimizing Preoperative Assessment Timing to Reduce Surgical Cancellations: A Quality Improvement Project

**DOI:** 10.7759/cureus.94177

**Published:** 2025-10-09

**Authors:** Muhammad Zaeem, Rabia Khalid, Talha Ahmed, Fakeha Tariq, Huma Amjad, Hassan Imtiaz, Rabia Asghar, Usamah Mazhar, Usama Shahbaz, Atizaz A Jan

**Affiliations:** 1 Anesthesia and Critical Care, District Headquarters Hospital, Faisalabad, PAK; 2 Trauma and Orthopaedics, Poole Hospital, University Hospitals Dorset, Poole, GBR; 3 General Surgery, Shaikh Zayed Hospital, Lahore, PAK; 4 Trauma and Orthopaedics, University Hospitals Dorset, Poole, GBR; 5 Endocrinology and Diabetes, Royal Bournemouth Hospital, University Hospitals Dorset, Bournemouth, GBR

**Keywords:** closed-loop audit, cost-effectiveness in surgery, elective surgical procedure, quality improvement (qi), surgical cancellation

## Abstract

Introduction

Elective surgery cancellations are a major cause of inefficiency in healthcare systems, leading to wasted operating theater time, increased costs, and longer waiting lists. Many cancellations are attributable to inadequate preoperative assessment or incomplete optimization, especially when assessments are performed immediately before surgery. Preoperative assessment clinics allow timely identification and management of comorbidities, ensuring patients are medically optimized and required investigations are complete before the day of surgery. This project aimed to evaluate whether introducing earlier preoperative assessments could reduce cancellations for elective surgeries.

Methods

A quality improvement project was conducted at a public sector District Headquarters Hospital in Punjab, Pakistan. In cycle 1 (November 2021-January 2022), preoperative assessments were performed ≤1 hour before surgery. In cycle 2 (December 2022-February 2023), assessments were conducted ≥24 hours before surgery. Data on elective surgical cases (orthopedics, general surgery, and ENT) were collected from anesthesia postponement logs. The primary outcome was the rate of postponements due to inadequate optimization or incomplete laboratory work-up, and wasted bed days per 100 patients. Chi-squared testing was employed, with *p* < 0.05 considered statistically significant.

Results

A total of 1,884 patients were included across the two cycles. During cycle 1, 1,129 cases were assessed, while in cycle 2, a total of 755 cases were evaluated. Sixty-nine patients (6.1%) were postponed during the time frame for cycle 1. In contrast, 23 patients (3.0%) experienced delays to theaters during cycle 2. This represented a relative risk reduction of 50%, with an absolute risk reduction of 3.07%, which was found to be statistically significant (p = 0.02). Wasted bed days per 100 patients decreased from 20.1 to 6.8.

Conclusion

Shifting preoperative assessments to at least 24 hours before surgery halved avoidable cancellations, reduced wasted bed days, and improved operating theater efficiency. This low-cost intervention is especially relevant for resource-limited health systems where theater efficiency and bed availability are critical.

## Introduction

Elective surgery cancellations are a global problem, with reported rates ranging from 10% to 20% worldwide and up to 40% in some low- and middle-income countries (LMICs) [1-3]. In Pakistan, studies have reported same-day cancellation rates as high as 18%-30%, with incomplete preoperative work-up and poorly controlled comorbidities as leading causes [4,5]. Such cancellations waste operating room time, delay care, and increase hospital costs and patient dissatisfaction.

Preoperative assessment clinics are designed to reduce this burden by identifying patient comorbidities, ensuring necessary laboratory and imaging work-up is complete, and optimizing patients in advance of surgery [6-8]. Evidence from high-income countries shows these clinics significantly reduce day-of-surgery cancellations and improve operating theater utilization [9,10]. For example, a large study in the United States found that implementing structured preoperative clinic assessments reduced same-day cancellations by nearly 50% [6].

Despite this, many public sector hospitals, especially in LMICs such as Pakistan, do not routinely conduct assessments before the day of surgery, often performing them minutes before anesthesia. This practice increases the risk of discovering uncontrolled diabetes, hypertension, or incomplete tests on the day of surgery, leading to avoidable postponements. Given the potential economic impact of surgical cancellations, reducing preventable cancellations is crucial for improving patient flow and reducing costs [11]. This quality improvement project aimed to assess the impact of introducing a preoperative clinic 24 hours before surgery on the rate of elective surgery cancellations due to inadequate optimization.

## Materials and methods

A two-cycle quality improvement project was carried out at District Headquarters Hospital, Faisalabad, Punjab, Pakistan. All patients aged 18-85 years scheduled for elective surgery under general, regional, or neuraxial anesthesia in the departments of orthopedics, general surgery, and ENT were included. Patients undergoing local anesthesia procedures or emergency surgeries or postponed for non-clinical reasons (equipment malfunction, theater logistics, and patient refusal) were excluded. The primary outcome measure was the number of delays to theaters.

The first cycle was conducted between November 2021 and January 2022, where preoperative assessments were performed ≤1 hour prior to surgery, reflective of existing practice. Following the findings of this cycle, a dedicated preoperative assessment clinic was introduced. This ensured that all patients listed for elective surgical procedures were assessed at least 24 hours prior to surgery. The aim was to minimize the rate of elective surgery cancellations due to inadequate optimization. This was implemented as a mandatory step for all elective surgery procedures. A second cycle was undertaken to evaluate the efficacy of these measures. This was conducted between December 2022 and February 2023.

Time-based convenience sampling was used. Data were collected from anesthesia postponement logs and anonymized at source. Data were stored on Microsoft Excel (Microsoft Corp., Redmond, WA, US) with patient identifiers removed for the purposes of maintaining confidentiality. Statistical analysis was performed on JASP software (Jeffreys’s Amazing Statistics Program, University of Amsterdam, Amsterdam, Netherlands). Results were compared between cycles using Chi-squared testing, with p < 0.05 considered significant. The primary outcome was the proportion of elective cases postponed due to inadequate optimization or incomplete lab investigations. Wasted bed days per 100 patients were calculated as

$W = \left( \frac{n}{N} \times d \right) \times 100$

where W is wasted bed days per 100 patients, n is the number of patients postponed from elective surgery over a given month, and d is the number of days wasted for every patient who was postponed from elective surgery, a constant, 3, since elective lists repeated twice weekly.

## Results

A total of 1,884 patients were included, with 1,129 cases evaluated during the first cycle (November 2021-January 2022) and 755 during the second cycle (December 2022-February 2023). Postponement rates for cycle 1 stratified based on individual months were 27/403 (6.69%) cases in November and 25/403 (6.20%) in December, while 17/323 (5.26%) were delayed in January. The total number of delays was 69/1,129, attributing an overall postponement rate of 6.1% during this time frame. Results are displayed in Table 1.

**Table 1:** Postponements during cycle 1 (Nov 2021–Jan 2022)

Month	Postponements (n)	Total patients (N)	Rate	Wasted bed days per 100 pts
November 2021	27	403	6.7%	20.1
December 2021	25	403	6.2%	18.6
January 2022	17	323	5.26%	15.8
Total	69	1,129	6.11%	-

Following the introduction of a preoperative clinic assessment, the subsequent second cycle demonstrated the following postponement rates, stratified for individual months: 5/222 (2.25%) in December, 11/251 (4.38%) in January, and 7/282 (2.48%) in February. The cumulative postponement rate for the second cycle was 23/755 (3.0%). These results are presented in Table 2. The reduction from 6.1% to 3.0% over postponement rates was statistically significant (p < 0.05), with an absolute risk reduction of 3.07% and a relative risk reduction of 50.15%. Statistical analysis is given in Table 3.

**Table 2:** Postponements during cycle 2 (Dec 2022–Feb 2023)

Month	Postponements (n)	Total patients (N)	Rate	Wasted bed days per 100 pts
December 2022	5	222	2.25%	6.8
January 2023	11	251	4.38%	13.1
February 2022	7	282	2.48%	7.4
Total	23	755	3.05%	-

**Table 3:** Statistical analysis (comparison between cycles 1 and 2) *Chi-squared value **p-value < 0.05 considered statistically significant

Cycle	Postponements (n)	Total patients (N)	Rate	X^2^*	p-value**	Absolute risk reduction	Relative risk
1	69	1,129	6.11%	-	-	-	-
2	23	755	3.05%	9.15	0.002	3.07%	0.50

## Discussion

This quality improvement project demonstrated that performing preoperative assessments at least 24 hours before surgery significantly reduced elective surgery postponements. The postponement rate decreased from 6.1% to 3.0%, representing a relative risk reduction of nearly 50%. These findings highlight the importance of early preoperative optimization in reducing preventable delays, particularly in resource-limited health systems.

Our results are consistent with prior work in LMICs, where incomplete investigations and poorly controlled comorbidities are leading causes of last-minute cancellations [1,3,4]. Similar to our findings, studies in Pakistan and other LMICs have shown that establishing structured preoperative pathways can reduce same-day cancellations by up to one-third [4,5,11]. At the same time, high-income country data demonstrate that preoperative clinics consistently improve operating room efficiency and reduce delays [6,7,9]. Ferschl et al. reported a 50% reduction in cancellations after the implementation of preoperative clinic visits [6], while Correll et al. showed that early evaluation identified perioperative risks otherwise missed during day-of-surgery assessments [7].

More recent studies further support these conclusions. Liu et al. reported from a large Chinese medical center that preoperative clinic attendance was strongly associated with reduced cancellations and improved perioperative safety [12]. Similarly, Lamperti et al. emphasized in European guidelines that structured preoperative evaluation should be a cornerstone of perioperative medicine, not only for medical optimization but also for system efficiency [9]. In addition to efficiency, patient-centered benefits are notable. Avoidable cancellations cause distress, financial loss, and logistical disruption for patients and families [3,13]. Randomized and prospective studies have shown that alternative models, such as telephone-based or digital preoperative assessments, can also lower day-of-surgery cancellations and may represent scalable options for resource-constrained settings [14-16].

By shifting assessments earlier, hospitals not only enhance throughput but also minimize patient dissatisfaction and mistrust in the health system. An important health system impact of reducing cancellations is the reduction of wasted bed days. Our study found a decrease from 20.1 to 6.8 bed days per 100 patients, consistent with prior reports on the economic consequences of cancellations [11]. The Iranian experience demonstrated that each cancelled case imposes significant costs on hospitals and patients [11], while similar analyses have estimated lost operating theater time as one of the most expensive forms of inefficiency in surgery [14,17]. For Pakistan’s public hospitals, where bed capacity and theater utilization are already stretched, such improvements could translate into substantial gains in access to timely surgery. This aligns with global surgical system strengthening goals that emphasize equity, timeliness, and quality of surgical care [18].

This study has several limitations. First, it is a single-center quality improvement project without randomization; residual confounding (changes in staffing, seasonal variation, or concurrent process improvements) may partly explain the observed effect. Second, the intervention targeted clinically preventable cancellations (incomplete work-up or unoptimized comorbidities) but did not address non-clinical causes such as equipment failure, emergency case intrusion, or patient no-shows-factors that can also drive cancellations in many settings. Third, our follow-up periods were short (three months per cycle); longer-term data are needed to assess sustainability and whether initial gains can be maintained at scale. Fourth, while we report reductions in wasted bed days, we did not perform a formal cost-effectiveness or budget-impact analysis; future work quantifying financial return on investment would help inform policy decisions.

## Conclusions

Introducing a structured preoperative clinic 24 hours before elective surgery reduced avoidable cancellations by 50% and significantly improved theater efficiency. This low-cost, high-impact intervention is especially relevant for resource-limited settings, with the potential for reducing healthcare costs and minimizing patient dissatisfaction at the same time. We recommend the use of such preoperative assessment clinics for all elective surgeries.
